# Carcinoembryonic Antigen (CEA) Elevation in Routine Screening Unveils Stage IV Lung Adenocarcinoma

**DOI:** 10.7759/cureus.83751

**Published:** 2025-05-08

**Authors:** Farha Munira Mohamed Kamel, Noor Azimah Muhammad, Amir Zharif Adenan

**Affiliations:** 1 Family Medicine, Universiti Kebangsaan Malaysia Medical Centre, Kuala Lumpur, MYS; 2 Public Health Medicine, Faculty of Medicine, Universiti Teknologi Majlis Amanah Rakyat (MARA) (UiTM), Sungai Buloh, MYS

**Keywords:** atypical presentation, cea, colonoscopy, lung adenocarcinoma, malignant pleural effusion

## Abstract

Carcinoembryonic antigen (CEA) is a tumor marker most often associated with colorectal cancer, but elevated levels may also indicate other malignancies, including lung cancer. Although not recommended as a standalone screening or diagnostic tool, persistent elevation in CEA levels may warrant further investigation. This case report describes a 71-year-old woman who was found to have progressively rising CEA levels during routine private health screening despite being asymptomatic. Initial workup, including colonoscopy, revealed no evidence of colorectal malignancy. However, further evaluation prompted by new respiratory symptoms and imaging findings led to the diagnosis of Stage IVa lung adenocarcinoma. This case underscores the importance of a comprehensive diagnostic approach in patients with unexplained tumor marker elevation. It highlights the potential role of primary care in identifying atypical presentations of serious conditions.

## Introduction

Carcinoembryonic antigen (CEA) is a nonspecific tumor marker not recommended as a cancer screening tool. CEA levels are expected to decline with age, and a continuous increase warrants thorough evaluation, particularly for gastrointestinal malignancies such as colorectal cancer. Elevated CEA levels are often associated with advanced or metastatic disease and are primarily used for monitoring cancer treatment response or post-treatment surveillance for recurrence [1,2]. However, its diagnostic specificity and sensitivity remain limited, and some practitioners have inappropriately used it for wellness screening. A CEA level above 2.9 ng/mL in asymptomatic, low-risk individuals does not necessarily indicate advanced gastrointestinal cancer with metastases. Elevated CEA levels may also be seen in lung cancer, non-malignant conditions such as acute and chronic inflammation, benign tumors, renal or hepatic insufficiency, and in smokers [3].

An isolated elevation in CEA poses a diagnostic challenge, particularly in an asymptomatic individual. A comprehensive clinical assessment, including imaging and molecular diagnostics, is crucial to determine the underlying cause. This case report provides insights into the diagnostic approach for nonspecific tumor marker elevations and highlights the role of personalized medicine in oncological management.

## Case presentation

A 71-year-old woman was found to have progressively elevated CEA levels during private health screening and follow-up. The initial CEA level was 48.6 ng/mL, which rose to 804.2 ng/mL over 12 months. Concurrently, two other tumor markers, cancer antigen (CA) 19-9 and CA 125, demonstrated an increasing trend (Table 1). Throughout this period, the patient remained asymptomatic. She was a known β-thalassemia carrier and had been on long-term treatment for hypertension and dyslipidemia. She had a history of premature menopause at the age of 44 following a total abdominal hysterectomy with bilateral salpingo-oophorectomy for uterine fibroids and ovarian cysts. She was a lifelong non-smoker, had no significant medical or family history of malignancy, and led an active, healthy lifestyle.

**Table 1:** Summary of the patient’s tumor marker trend CEA: carcinoembryonic antigen, CA: cancer antigen

CEA level (ng/mL)	CA 19-9 (U/mL)	CA 125 (U/mL)
0-5	0-37	0-35
48.6	-	-
123.0	23.0	16.0
145.5	-	-
571.8	-	-
754.9	28.0	113.0
804.2	-	-

**Figure 1:**
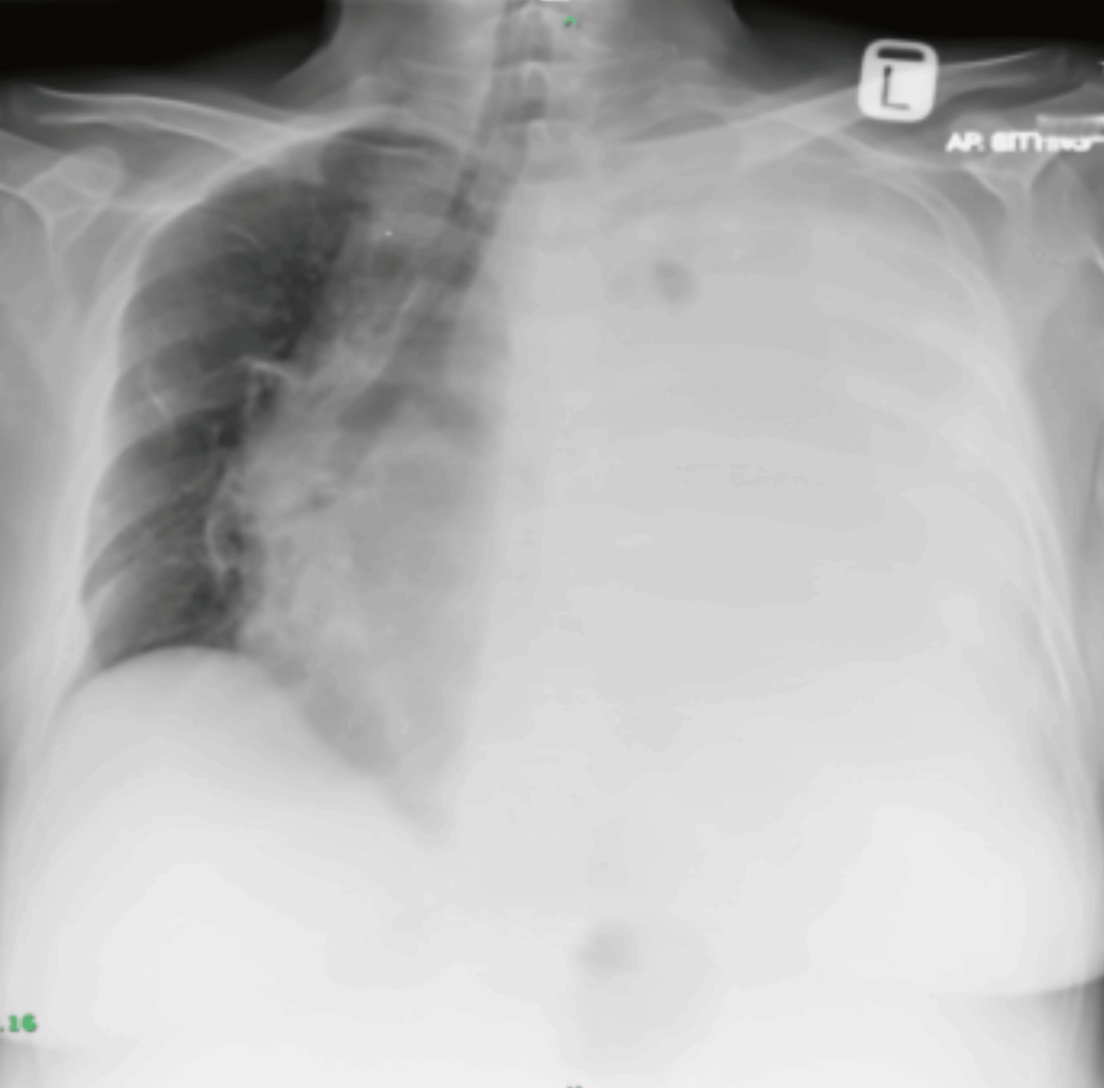
Chest radiograph showing a wiped-out left lung with tracheal and mediastinal shift to the right

Given the significant elevation in CEA, she was referred to a tertiary center with an initial clinical suspicion of colorectal carcinoma. Colonoscopy revealed only benign findings, including colonic diverticulosis and a small hyperplastic polyp, with no evidence of malignancy. While undergoing further investigations, the patient began to experience shortness of breath, and examination revealed oxygen saturation of 90% on room air. A chest radiograph (Figure 1) and CT scan of the thorax showed a massive left-sided pleural effusion, a right upper lobe lung nodule, and mediastinal lymphadenopathies.

**Figure 2:**
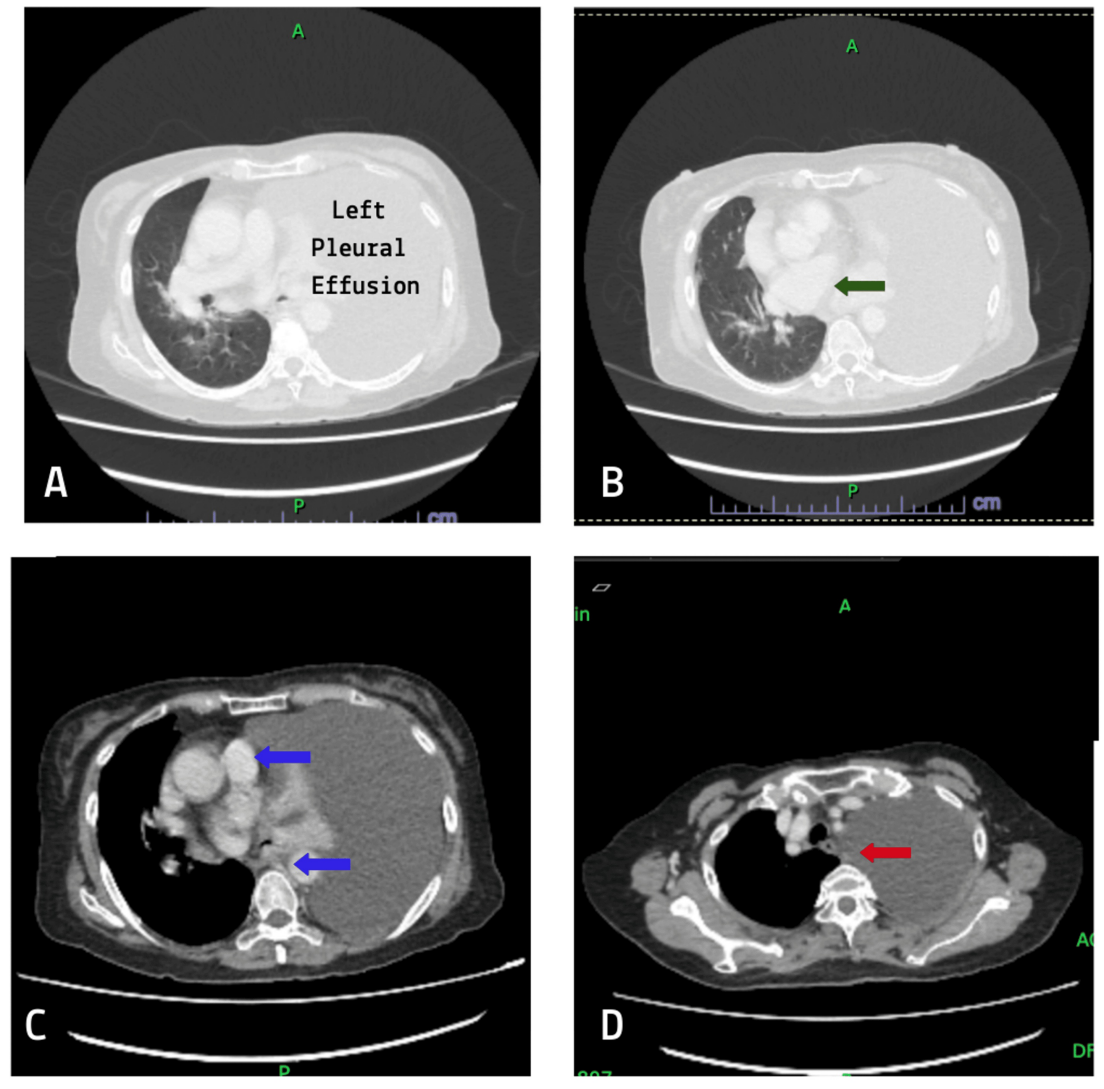
Axial CT scans of the thorax demonstrating a large left pleural effusion with passive compression of the underlying lung (A), mediastinal shift to the right due to mass effect from the effusion (green arrow, B), prominent mediastinal lymphadenopathy suggestive of possible lymph node metastases (blue arrows, C), and a small, indeterminate right upper lobe pulmonary nodule (red arrow, D) CT: computed tomography

A CT-guided biopsy of the lung nodule along with pleural fluid cytology confirmed the diagnosis of lung adenocarcinoma (Figure 2). Immunohistochemical staining of the samples demonstrated thyroid transcription factor-1 (TTF-1) and cytokeratin 7 (CK7) positivity. Molecular analysis revealed the presence of an epidermal growth factor receptor (EGFR) exon 19 deletion mutation. The disease was staged as stage IVa (T1cN3M1a) lung adenocarcinoma, characterized by a small primary lung lesion with extensive lymphatic and intrathoracic metastasis. Management was focused on symptomatic relief and targeted therapy. A temporary indwelling pleural catheter (IPC) was inserted to alleviate dyspnea, and treatment with gefitinib, an EGFR tyrosine kinase inhibitor, was initiated.

## Discussion

This case highlights the diagnostic challenges and clinical importance of monitoring tumor markers, particularly CEA, in asymptomatic patients undergoing wellness health screening. Apart from being linked to colorectal cancer, CEA may be elevated in lung, gastric, and pancreatic cancers [4]. However, due to its limited sensitivity and specificity (48.2% sensitivity, 93.8% specificity), CEA is not recommended as a stand-alone screening tool [5,6]. Its primary clinical utility lies in monitoring disease progression or recurrence [7].

The concurrent elevation of CA-125 and CA 19-9 in this patient further supported the presence of malignancy, prompting more detailed investigations. Nonetheless, using tumor markers such as CEA, CA 19-9, or CA-125 as exclusive tools for lung cancer screening is not recommended. Elevated CEA in lung adenocarcinoma helps support diagnosis and monitor disease course but should not be relied upon for screening [8,9]. While CA 19-9 is commonly associated with gastrointestinal malignancies [10,11] and CA-125 with ovarian cancers, both markers have limited roles in diagnosing lung cancer [11].

Although combining these markers may improve sensitivity, the issue of specificity remains, making them more suitable for prognostication, treatment monitoring, and recurrence detection rather than for primary diagnosis [12]. CEA, in comparison, has demonstrated higher specificity and is more reliable than CA-125 in the context of malignant pleural effusions [13,14]. When both CEA and CA-125 are elevated, the likelihood of malignant pleural effusion increases significantly, with one study reporting a 105-fold increase in risk [13].

The initial suspicion of colorectal cancer based on progressively rising CEA levels was reasonable but was ruled out following benign colonoscopy findings. Subsequent chest radiograph and CT of the thorax revealed evidence suggestive of lung cancer. These findings reinforce the necessity of integrating imaging modalities into the diagnostic workup of unexplained tumor marker elevations. Combining imaging and tumor markers can help detect subclinical disease, aid in risk stratification, and prevent unnecessary interventions [15,16].

The final diagnosis of lung adenocarcinoma was confirmed through cytopathological examination and immunohistochemical positivity for TTF-1 and CK7 on pleural fluid and tissue biopsy. The detection of an EGFR exon 19 deletion mutation enabled targeted therapy with gefitinib, an EGFR tyrosine kinase inhibitor, highlighting advancements in precision oncology [17]. This personalized approach and symptomatic relief through an IPC improved the patient’s quality of life and prognosis.

## Conclusions

This case emphasizes the importance of maintaining a high index of suspicion for malignancy in elderly patients, even in the absence of classic symptoms and when initial investigations are inconclusive. It underscores the value of a comprehensive diagnostic approach integrating tumor markers, imaging modalities, and molecular diagnostics. Molecular profiling, as demonstrated by identifying the EGFR mutation and the subsequent use of gefitinib, exemplifies the critical role of personalized medicine in improving outcomes for patients with advanced-stage malignancies.
